# P-237. The CLABSI (Central Line Audit by Staff Investigators) Intervention: Reducing Central Line Use in a Medical ICU using Physician-Led Prospective Audit and Feedback of Catheter Necessity

**DOI:** 10.1093/ofid/ofae631.441

**Published:** 2025-01-29

**Authors:** Ken Dekitani, Michael Ben-Aderet, Meghan Madhusudhan

**Affiliations:** Cedars-Sinai Medical Center, Los Angeles, California; Cedars Sinai Medical Center, Los Angeles, CA; Cedars-Sinai Medical Center, Los Angeles, California

## Abstract

**Background:**

Prompt removal of unnecessary central lines has been associated with reduction in central line associated bloodstream infections (CLABSI). Following the COVID-19 pandemic, sustained increases in central line utilization and CLABSI were seen at our medical ICU (MICU). Here we present a quality improvement intervention of a weekly physician-led audit and feedback to reduce central line utilization.

**Methods:**

The intervention was conducted in the MICU at a single tertiary-care hospital from June 2023 to February 2024. Once weekly, 2 physicians did an electronic medical record audit of all central lines. Line necessity was defined as requiring one or more of the following: irritant medication use, renal replacement therapy, hemodynamic monitoring, total parenteral nutrition, or inability to obtain peripheral access. Lines for renal replacement therapy (HD lines) were excluded from evaluation as they were medically necessary by definition. Once an unnecessary line was identified, the primary ICU fellow and attending were sent a text message prior to morning rounds to recommend removal. Central line removal rates and patient demographic data were collected from June 2023 to February 2024. Data on central line utilization and CLABSI rates were collected from July 2023 to December 2023 and compared to data from January 2022 to June 2023.

**Results:**

365 patients and 447 central lines were reviewed. 49 (11%) central lines were deemed unnecessary and removal recommended, of which 26 (53%) were removed within 24 hours. The central line device utilization ratio (DUR) in line days per patient days decreased by 20%, from 0.48 to 0.38 [p = < 0.0001]. When HD lines were included, we observed a reduction in DUR by 8%, from 0.61 to 0.56 [p = < 0.0001]. The CLABSI National Healthcare Safety Network Standardized Infection Ratio (SIR) pre-implementation was 2.84 and post-implementation was 2.83 [p = 1.00].

**Conclusion:**

A physician-led, once weekly prospective audit and feedback of central line necessity led to a substantial decline in central line utilization rates in our medical ICU. There was no observed reduction in CLABSI SIR, however this may be due to the short intervention period and low incidence rate of CLABSI; over time it would be expected that a reduction in central line days would lead to a reduction in CLABSI.

**Disclosures:**

**All Authors**: No reported disclosures

